# Supplementary material for: Crystalline TiO2 protective layer with graded oxygen defects for efficient and stable silicon-based photocathode
Source: Nat Commun. 2018 Sep 3;9:3572. doi: 10.1038/s41467-018-05580-z (PMC6120862; doi:10.1038/s41467-018-05580-z)
Supplement: Supplementary file 1 — Supplementary Information [file 41467_2018_5580_MOESM1_ESM.pdf]

Supplementary Information

Crystalline TiO_2 protective layer with graded oxygen defects for efficient and stable silicon-based photocathode

Zheng et al.

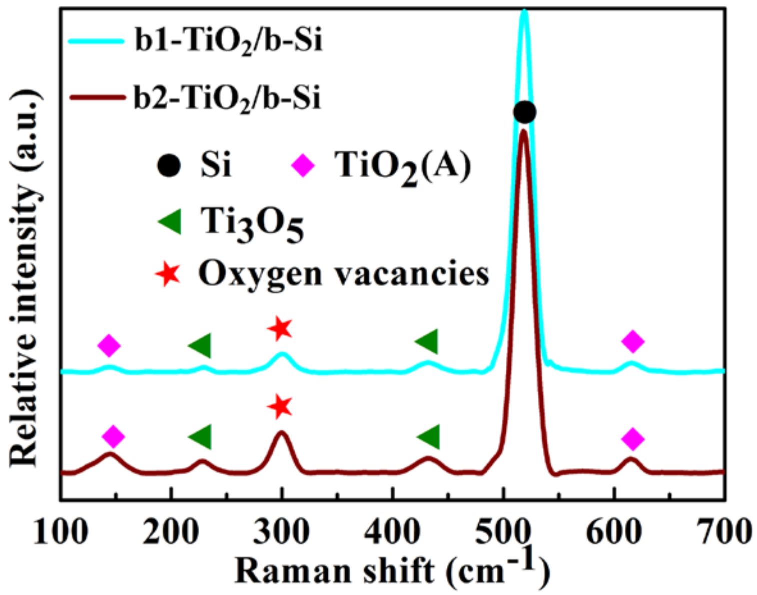

Supplementary Figure 1. Raman spectra of b1-TiO_2/b-Si (Cambridge blue line) and b2-TiO_2/b-Si (brown line). Raman measurement was carried out using a HR800 Raman microscope instrument with 532 nm Ar ion laser and a resolution of 1 cm^{-1}.

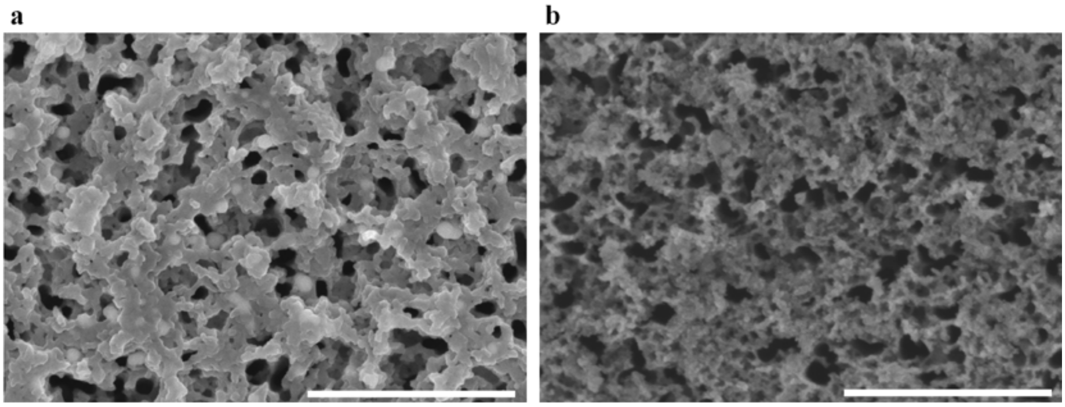

Supplementary Figure 2. Top view FESEM images of the samples. **a** FESEM image of b-Si. **b** FESEM image of b2-TiO_2/b-Si. The scale bar labels are 500 nm.

Aiming to determine the microstructure of the film, field emission scanning electron microscopy (FESEM) was employed to observe the surface and cross section of the film.

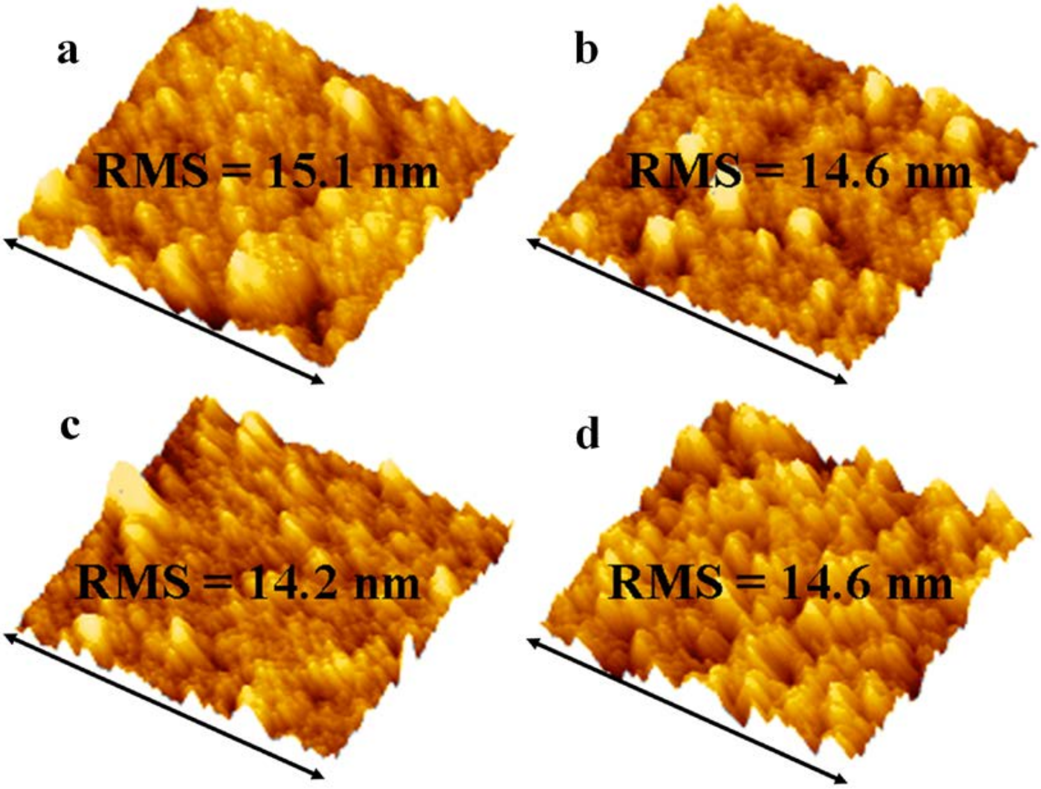

Supplementary Figure 3. AFM images and root mean square (RMS) roughness of the samples. **a** AFM image of b-Si. **b** AFM image of c-TiO_2/b-Si. **c** AFM image of b1-TiO_2/b-Si. **d** AFM image of b2-TiO_2/b-Si. The scale bar labels (double sided arrow) are 2000 nm.

The surface morphology of the film was obtained by an atomic force microscope (AFM, SII Nano Technology Ltd., Nanonavi Π) with noncontact mode.

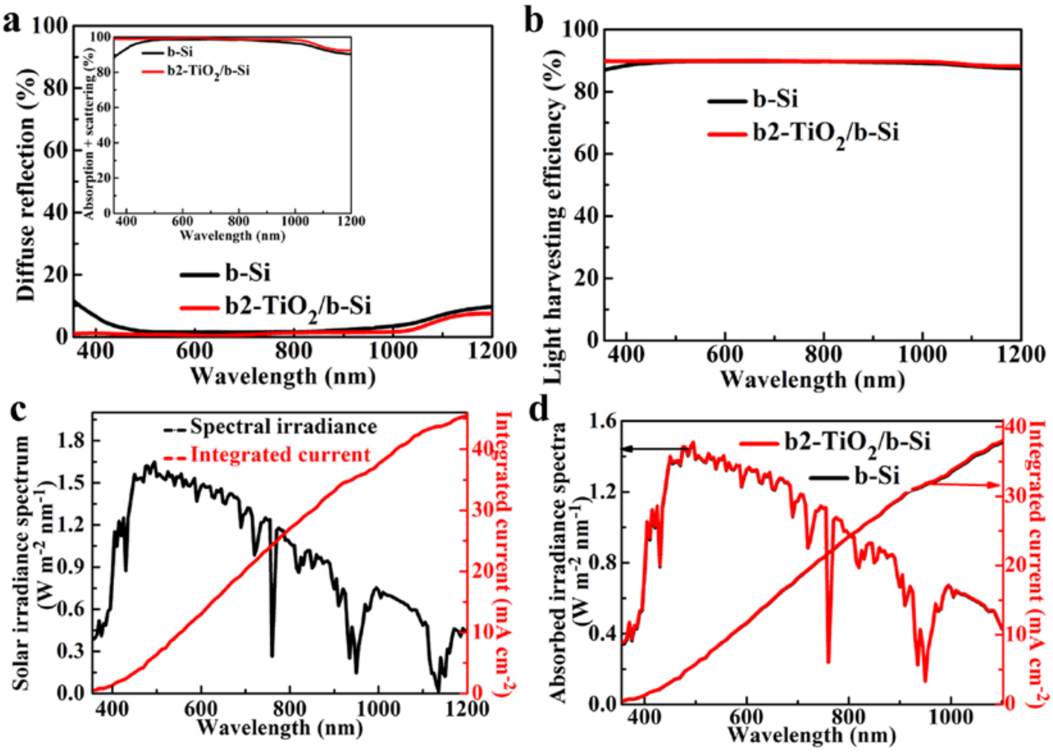

Supplementary Figure 4. Optical properties of the samples. **a** UV-visible reflectance of b-Si and b2-TiO_2/b-Si in air. The inset is UV-visible absorption spectra of b-Si and b2-TiO_2/b-Si derived from reflectance spectra through the relation absorption = 100 – transmittance – reflectance, assuming zero transmittance. **b** The light harvesting efficiencies (η_{LHE}) of b-Si and b2-TiO_2/b-Si. η_{LHE} was calculated from the absorbance data (η_{ABS}) shown in Figure S4a via the equation η_{LHE} = $(1\text{-}10^{-\eta ABS})$ × 100%. **c** Irradiance and integrated current of AM 1.5G solar spectrum. **d** Absorbing solar irradiance spectra and integrated current of b-Si and b2-TiO_2/b-Si. The absorbing solar irradiance spectra were the product of the AM 1.5G irradiance and η_{LHE}. Photocurrent density (J_{abs}) under 100% absorbed photon conversion efficiency can be estimated by the integrated current at the photocathodes across 350-1100 nm wavelength range.

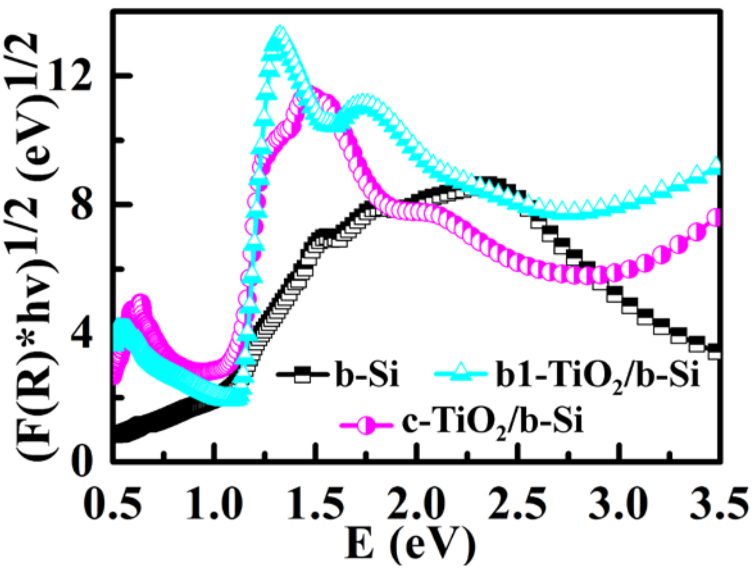

Supplementary Figure 5. The optical absorption coefficient as a function of the incident photon energy for indirect allowed transition for (black square) b-Si, (pink circle) c-TiO_2/b-Si and (Cambridge blue triangle) b1-TiO_2/b-Si.

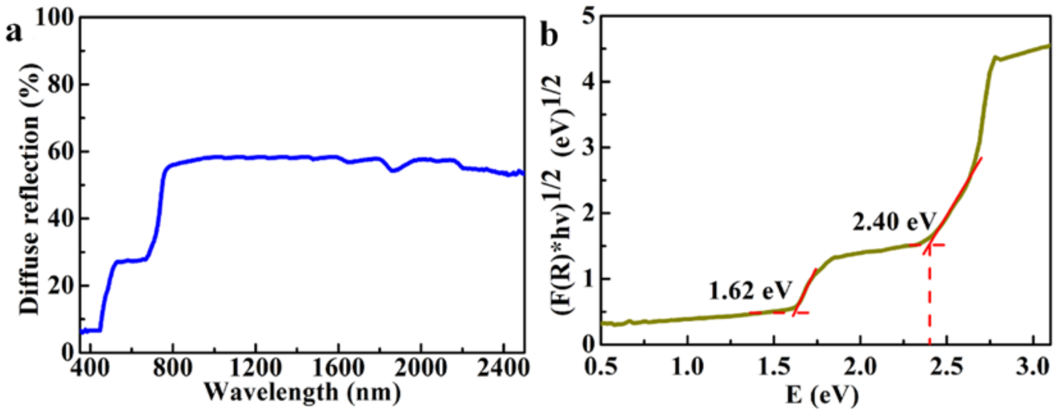

Supplementary Figure 6. Optical properties of black TiO_2 film. **a** Diffuse reflection spectrum. **b** plot of optical absorption coefficient vs incident photon energy of black TiO_2 film deposited on BK7 wafer.

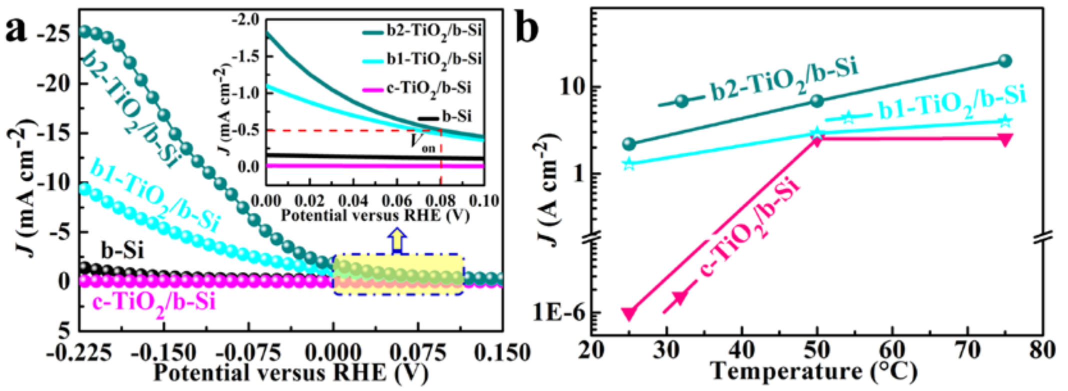

Supplementary Figure 7. PEC and electronic measurements of Si-based photocathodes. **a** *J-V* curves (scan rate is 0.01 V·s^{-1}) in 1.0 M NaOH under 1 sun illumination. The current density measured in darkness is almost horizontal line, namely 0 mA·cm^{-2}. The inset is the high maginification image corresponding to the marked area. **b** Temperature-dependent current density measurement through b2-TiO_2/b-Si (purple), b1-TiO_2/b-Si (green), and c-TiO_2/b-Si (pink). Current measured in a probe station (no electrolyte present) at a Si substrate voltage vs the Ag contact of 1.0 V. Current-voltage data are provided in Supplementary Fig. 10.

Supplementary Fig. 7a presents photocurrent density-potential (*J*-*V*) curves of the four samples with simulated solar irradiation at 1 sun (AM 1.5G) in basic solution. All photoelectrodes showed negligible current versus the potential range in the dark. Under illumination, the b-Si had an onset of photocurrent (0.5 mA·cm^{-2}) at -0.152 V vs RHE. On depositing the crystal TiO_2 layer, a visible quenching in PEC activity was observed because the electron transfer was blocked. Interestingly, b1-TiO_2/b-Si and b2-TiO_2/b-Si by vacuum annealing showed the onsets shifted to ~0.069 and ~0.080 V, respectively. Additionally, hydrogen evolution current densities are ~1.8 and ~1.1 mA·cm^{-2} at the reversible potential (0 V vs RHE) for b1-TiO_2/b-Si and b2-TiO_2/b-Si, respectively. The limiting current density of b2-TiO_2/b-Si was about 25.2 mA·cm^{-2}, which is obviously higher than that of b1-TiO_2/b-Si (about 9.3 mA·cm^{-2}).

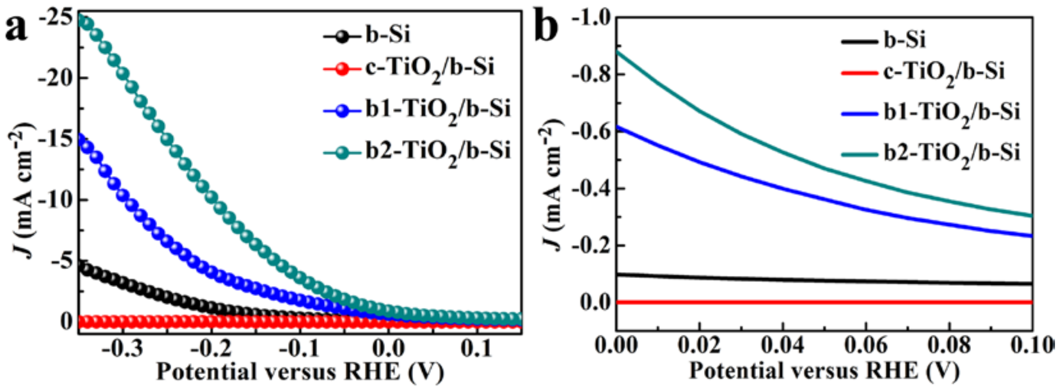

Supplementary Figure 8. PEC performance of the samples in the acid electrolyte. **a** *J*-*V* data of the samples with scan rate of 0.01 V·s^{-1} in 0.5 M H_2SO_4 under 1 sun illumination. The current density measured in darkness is almost horizontal line, namely 0 mA·cm^{-2}. **b** High magnification image corresponding to the potential from 0 V to 0.1 V.

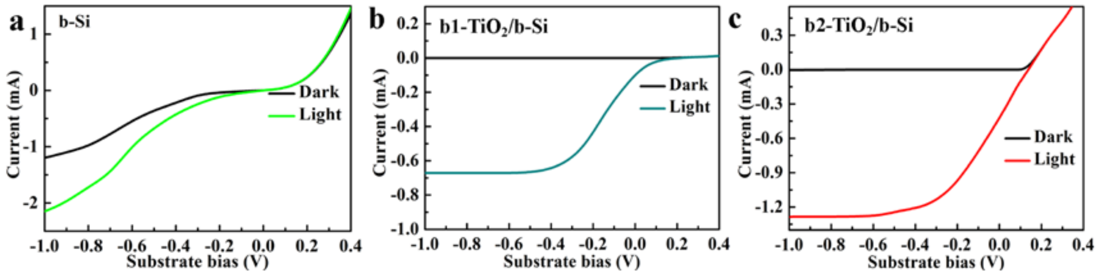

Supplementary Figure 9. Dry current-potential (*I*-*V*) measurements. **a** The *I*-*V* curves of b-Si before and after illumination. **b** The *I*-*V* curves of b1-TiO_2/b-Si before and after illumination. **c** The *I*-*V* curves of b2-TiO_2/b-Si before and after illumination. The measurements were conducted under 100 mW·cm^{-2} white light illumination for one metal silver dot on the samples

Current versus Voltage (*I*-*V*) curves were performed using a GPS150 probe station equipped with a HW-1616 constant temperature controller and a keithley 2400 digital source meter. The Xe lamp with AM 1.5G filter were used for illumination, and calibrated to AM 1.5 intensity (100 mW·cm^{-2}) through light power meter (PerfectLight Co. Ltd., PL-MW 200). Before the measurement of the light current, each sample was cut into 5 × 5 mm^2 and then glued onto one quartz glass by conductive silver paint (Leitsilber 200). Part of dried silver paint on the glass, which was exposed to air, is functioned as one electrode, while another electrode is produced by dotted the silver paint on surface of each sample. Tungsten probes were used to contact a front and back collectors for dry measurements. To obtain the light current, the voltage range was all set at -1.0 V ~0.4 V with or without irradiation.

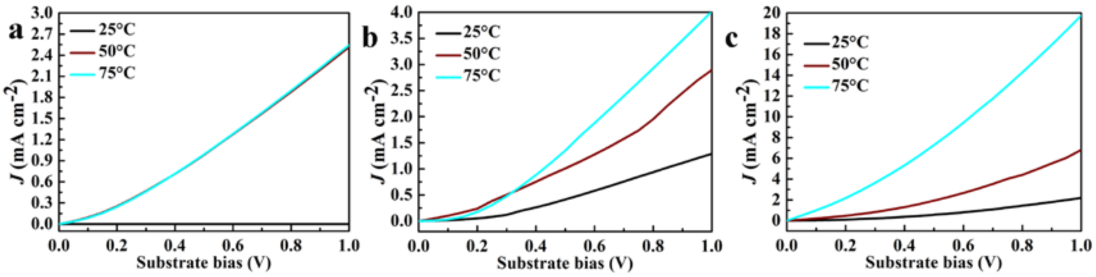

Supplementary Figure 10. Temperature dependent tunneling current measurement of the samples. **a** *J*-*V* curves of c-TiO_2/b-Si with the different temperature. **b** *J*-*V* curves of b1-TiO_2/b-Si with the different temperature. **c** *J*-*V* curves of b2-TiO_2/b-Si with the different temperature. Measurements were taken at (black line) 25, (brown line) 50 and (Cambridge blue line) 75 °C, respectively.

Electron tunneling through b1-TiO_2/b-Si and b2-TiO_2/b-Si was discovered at room temperature, in which the tunneling current of b2-TiO_2/b-Si is larger than that of b1-TiO_2/b-Si. The currents for both samples had some dependence on temperature, an indication of a thermally-activated conduction mechanism such as trap-assisted tunneling. From the *I*-*V* measurement, the resistance of b2-TiO_2/b-Si was estimated to be less than 0.5 $\Omega\cdot cm^2$ on b-Si substrate at room temperature. The complete dependence on the temperature of the electronic conduction from c-TiO_2/b-Si agrees with the thermally activated mode.

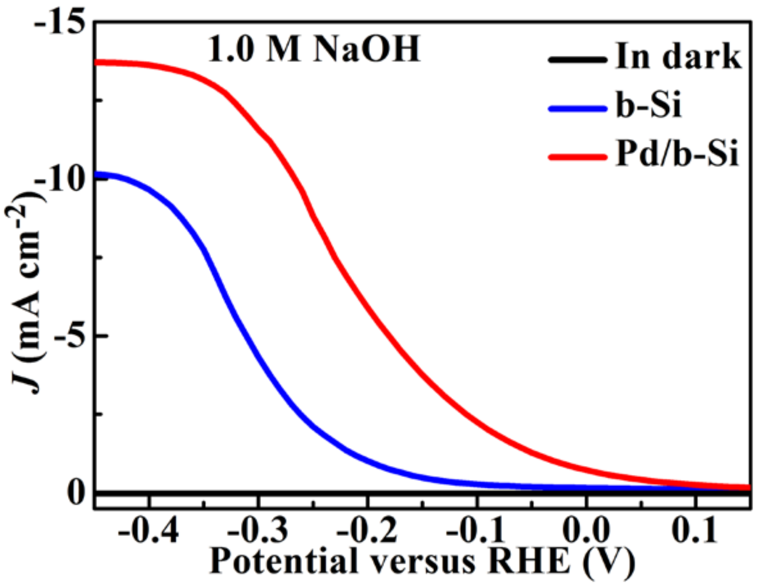

Supplementary Figure 11. *J*-*V* curves of b-Si and Pd/b-Si with scan rate of 0.01 V·s^{-1} in 1.0 M NaOH under 1 sun illumination. Here, higher applied potentials was used to obtain the limiting photocurrent density of b-Si and Pd/b-Si.

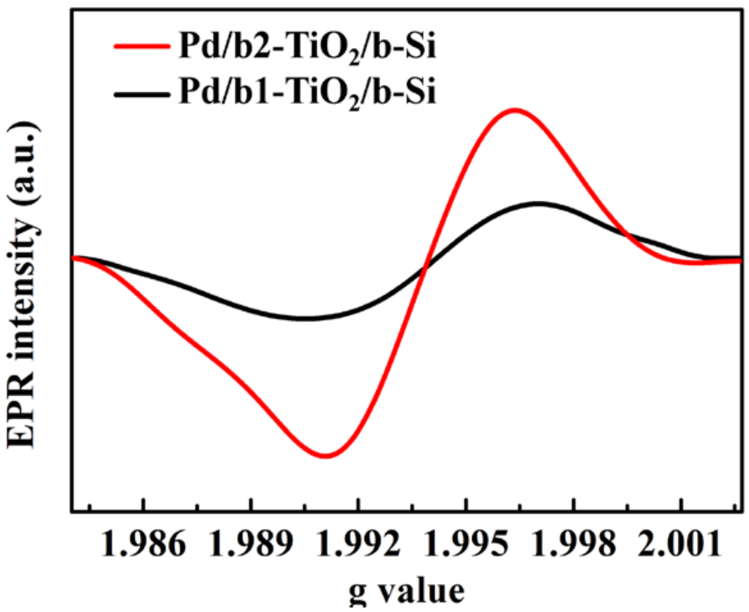

Supplementary Figure 12. EPR spectra of Pd/b1-TiO_2/b-Si (black line) and Pd/b2-TiO_2/b-Si (red line) at room temperature.

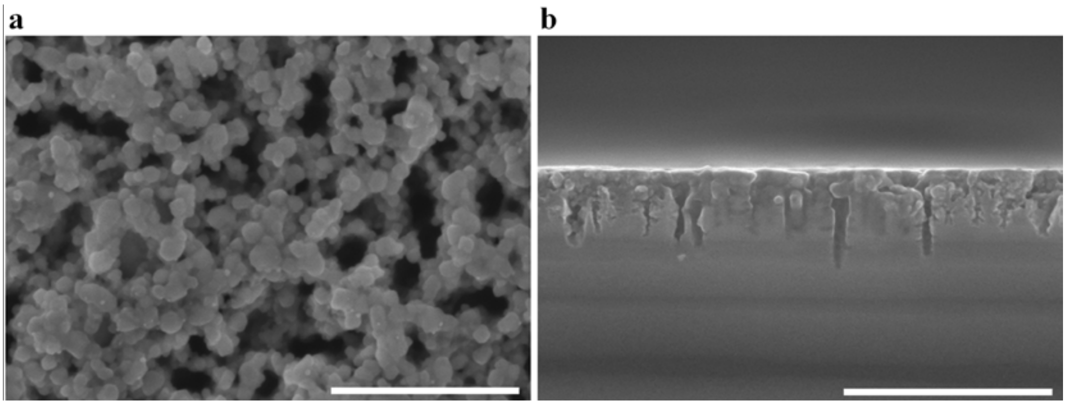

Supplementary Figure 13. FESEM images of Pd/b1-TiO_2/b-Si. **a** Top view FESEM image. **b** cross-sectional FESEM image. The scale bar labels are 300 and 500 nm in top view image and cross-sectional image, respectively.

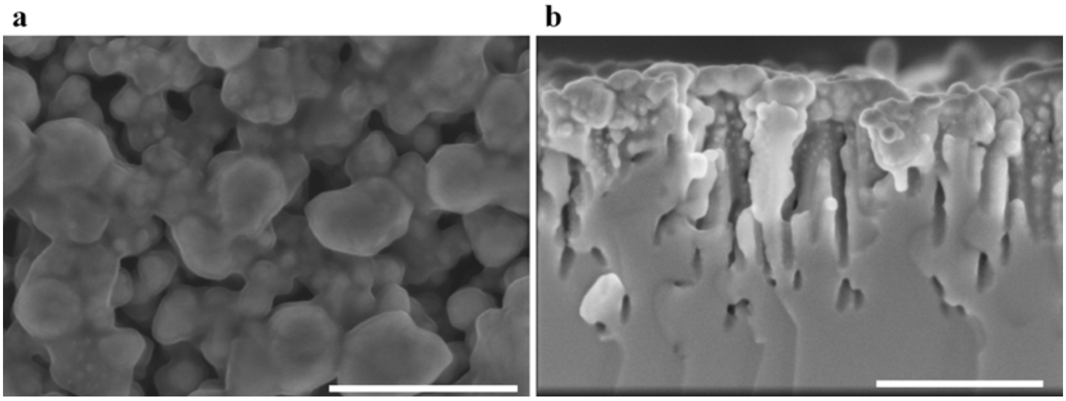

Supplementary Figure 14. FESEM images of Pd/b2-TiO_2/b-Si. **a** Top view FESEM image. **b** cross-sectional FESEM image. The scale bar labels are 300 and 400 nm in top view image and cross-sectional image, respectively.

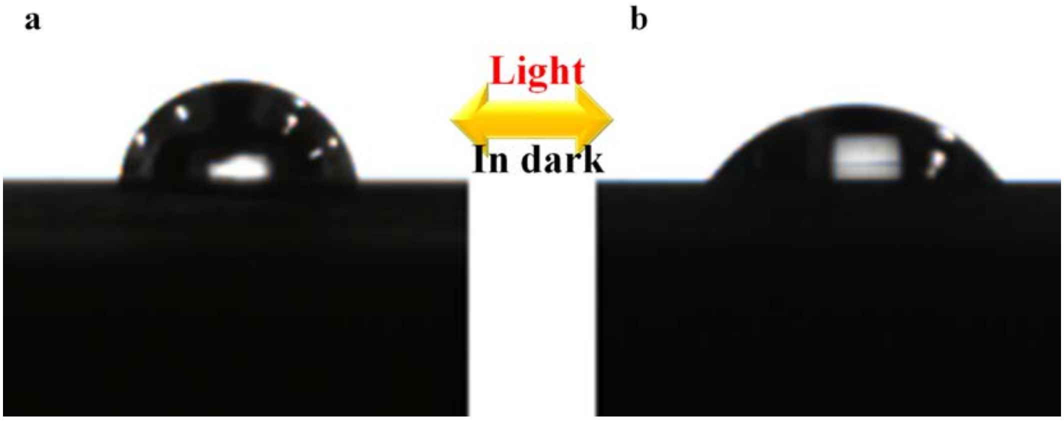

Supplementary Figure 15. Wettability of Pd/b2-TiO_2/b-Si. **a** Photographs of the spherical water droplets with changeable contact angles before illumination. **b** Photographs of the spherical water droplets with changeable contact angles after illumination.

Furthermore, the water droplet shape on Pd/b2-TiO_2/b-Si after UV-visible light illumination was still semielliptical (Supplementary Fig. 15), meaning the normal hydrophilicity (CA > 10°). Compared to b2-TiO_2/b-Si, the wettability variation of Pd/b2-TiO_2/b-Si result from the coverage of highly dispersed Pd nanoparticles.

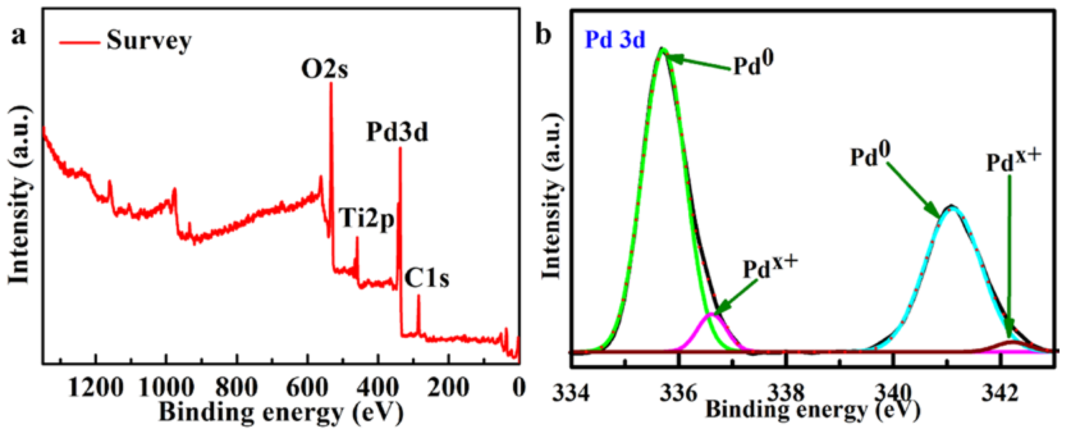

Supplementary Figure 16. XPS data of Pd/b2-TiO_2/b-Si. **a** XPS survey spectrum. **b** Pd 3d spectrum for the surface.

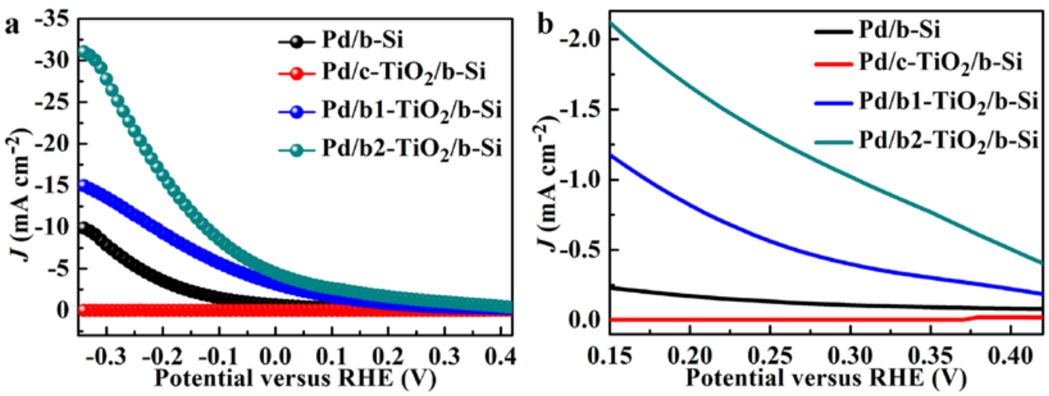

Supplementary Figure 17. PEC performance of the samples in the acid electrolyte. **a** *J*-*V* curves of the samples with scan rate is 0.01 V·s^{-1} in 0.5 M H_2SO_4 under 1 sun illumination. The current density measured in darkness is almost horizontal line, namely 0 mA·cm^{-2}. **b** High magnification image corresponding to the potential from 0.15 V to 0.45 V.

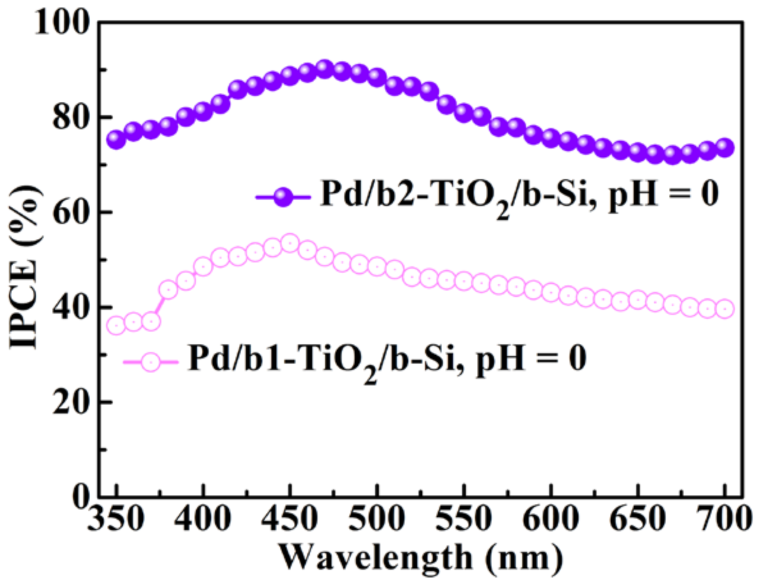

Supplementary Figure 18. IPCE of Pd/b1-TiO_2/b-Si and Pd/b2-TiO_2/b-Si in 0.5 M H_2SO_4 at -0.35 V vs RHE.

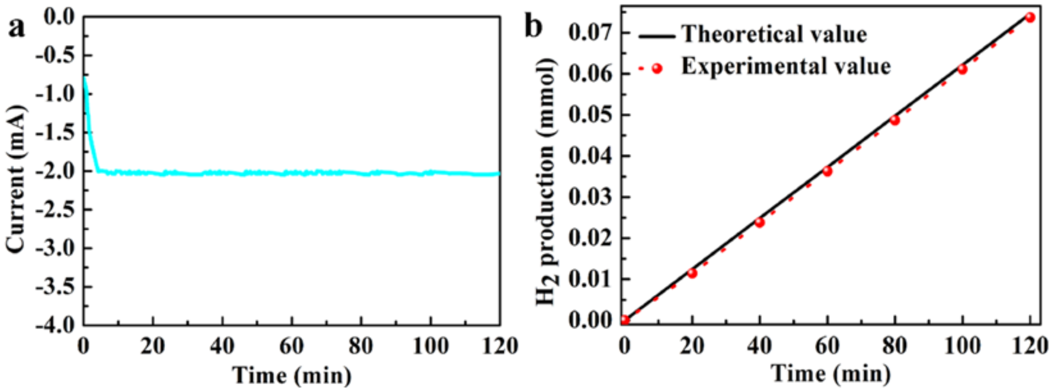

Supplementary Figure 19. Hydrogen production of Pd/b2-TiO_2/b-Si. **a** *I-t* plot of Pd/b2-TiO_2/b-Si held at -0.078 V vs RHE in 1.0 M NaOH under 1 sun illumination. **b** Photoelectrochemical hydrogen production for Pd/b2-TiO_2/b-Si via calculating and measuring in test conditions.

A gas-tight electrochemical set-up was applied to collect and measure the increased volume caused by H_2 production with a volumetric pipette. The volumes of set-up and aqueous solution electrolyte were 235 and 90 ml, respectively. The collected gas was further measured by gas chromatography (Lunanruihong Co. Ltd., SP-7820) with a CarbxenTM 1010 PLOT column and a thermal conductivity detector. The photocurrent at constant external bias of 79 mV vs RHE and gas product was further shown in Supplementary Fig. 19b.

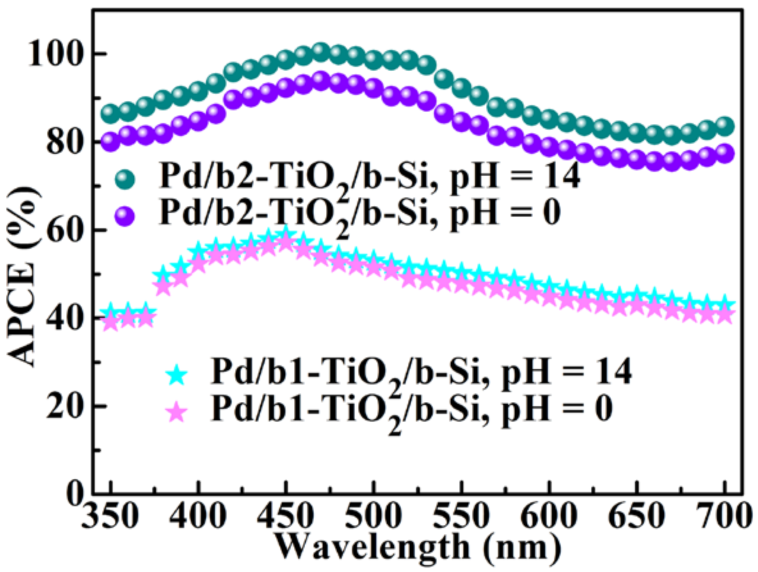

Supplementary Figure 20. Absorbed photon-to-current efficiency (APCE) of Pd/b1-TiO_2/b-Si and Pd/b2-TiO_2/b-Si in 1.0 M NaOH and 0.5 M H_2SO_4.

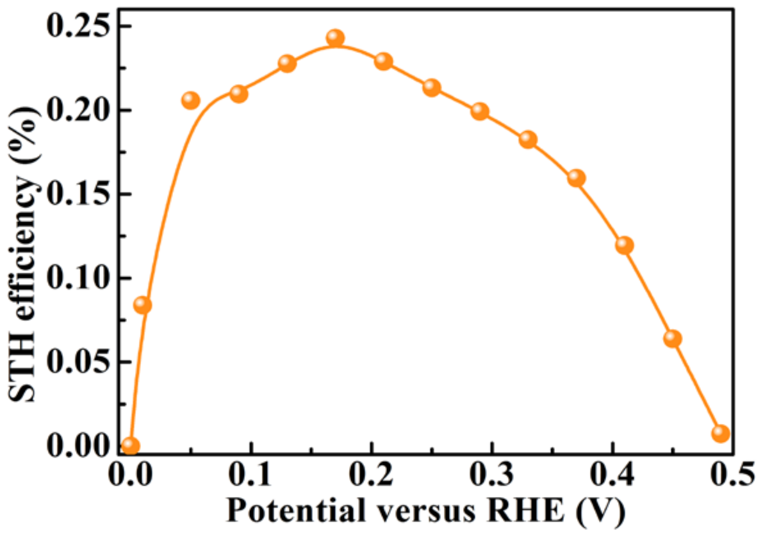

Supplementary Figure 21. Solar-to-hydrogen (STH) efficiency of Pd/b2-TiO_2/b-Si as a function of applied potential from Fig. 5a. The efficiency, depicted as η_{STH}, is calculated through

$$\eta_{STH} = \frac{1.23 \times (V\ vs\ RHE) \times J \times FY}{P_{incident}} \quad (1)$$

where V is the applied potential vs RHE, J the photocurrent density, FY the Faradaic yield, and $P_{incident}$ the illumination power density (100 mW·cm^{-2}). A maximum half-cell η_{STH} (~0.24%) of Pd/b2-TiO_2/b-Si is lower than the other n^+p-Si photocathodes, which can be attributed to the absence of p-n junctions.

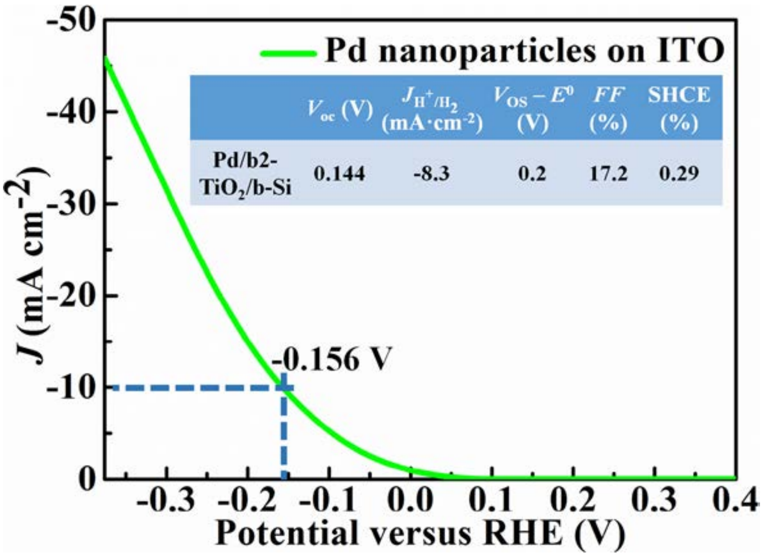

Supplementary Figure 22. Electrochemical characterization of Pd nanoparticles deposited on ITO in a 1.0 M NaOH electrolyte in dark. The inset is the characteristics of Pd/b2-TiO_2/b-Si photocathode in a 1.0 M NaOH electrolyte under simulated AM 1.5 G illumination. V_{OC} is the open-circuit voltage of the photocathode; V_{OS} is the potential measured at a water reduction current density of 1 mA·cm^{-2}; E^0 is the equilibrium water reduction potential in the 1.0 M NaOH electrolyte, which is 0 V vs RHE; J_{H^+/H_2} is the current density at E^0; FF is the fill factor of the photocathode; and SHCE is the solar-to-hydrogen conversion efficiency of the photocathode.

The preparation conditions of Pd nanoparticles on ITO are identical to those in b2-TiO_2/b-Si. The V_{OC} is calculated as follows:

$$V_{OC} = V_{ph,10} - V_{ca,10} \tag{2}$$

where $V_{ph,10}$ is the potential of Pd/b2-TiO_2/b-Si photocathode at a current density of 10 mA·cm^{-2} under illuminated, and $V_{ca,10}$ the potential of Pd nanoparticles on ITO at a current density of 10 mA·cm^{-2} in dark. The SHCE is calculated as follows:

$$\mathrm{SHCE} = \frac{\left|V_{OS} - E^0\right| J_{H^+/H_2} FF}{P_{incident}} \tag{3}$$

where $P_{incident}$ is the illumination power density.

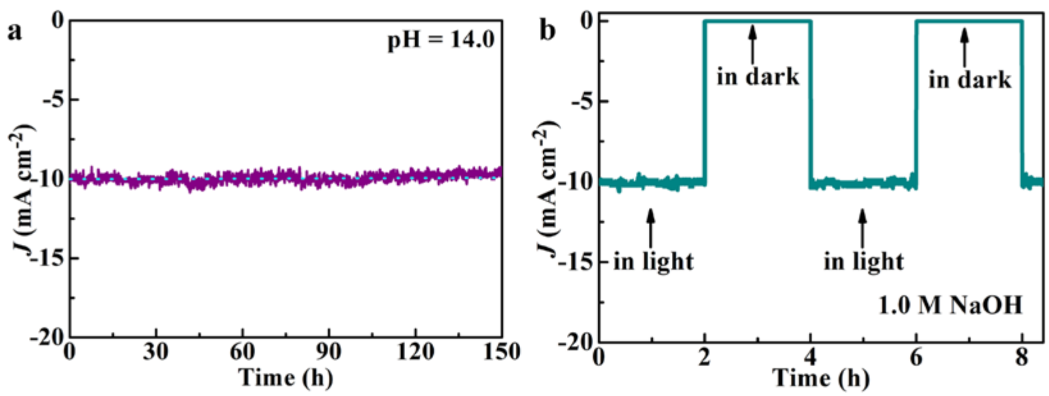

Supplementary Figure 23. Stability of Pd/b2-TiO_2/b-Si photocathode. **a** *J*-*t* plots of Pd/b2-TiO_2/b-Si held at -0.012 V vs RHE in 1.0 M NaOH under 1 sun illumination. **b** The variations of photocurrent density of Pd/b2-TiO_2/b-Si at -0.012 V vs RHE in 1.0 M NaOH by chopping light illumination.

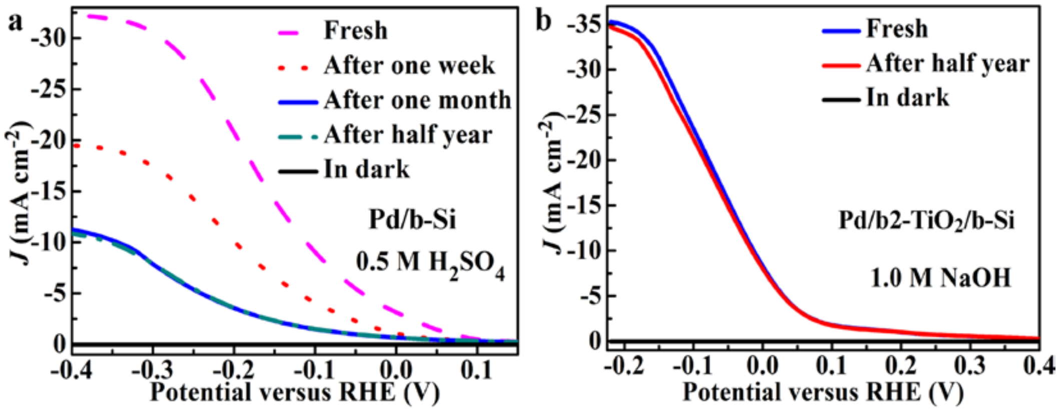

Supplementary Figure 24. PEC performance of the samples under different conditions. **a** Liner-sweep voltammetry (*J*-*V*) curves of fresh, one-week atmosphere-aged, one-month atmosphere-aged and half-year atmosphere-aged Pd/b-Si measured in 0.5 M H_2SO_4 electrolyte under one sun illumination. The silicon oxide layer is relatively stable in 0.5 M H_2SO_4 solution. **b** *J*-*V* curves of fresh and half-year atmosphere-aged Pd/b2-TiO_2/b-Si measured in 1.0 M NaOH electrolyte under one sun illumination. The nearly equivalent behaviors between fresh and half-year atmosphere-aged Pd/b2-TiO_2/b-Si was identified, evidencing the good in-air stability.

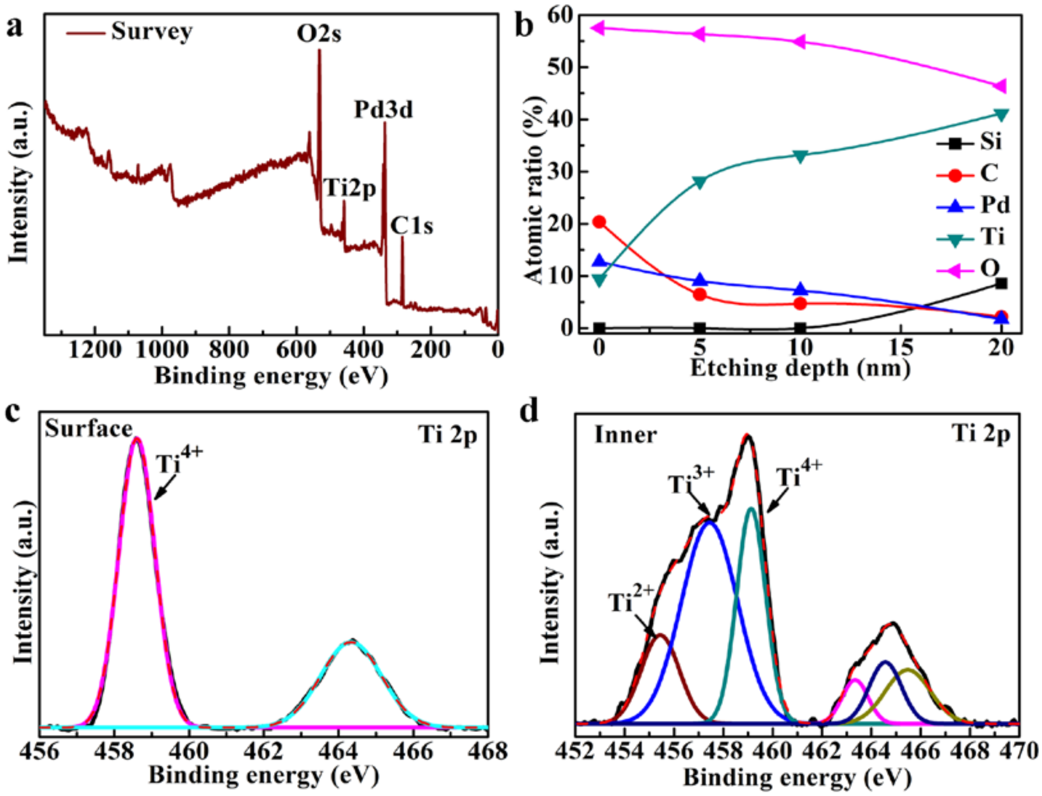

Supplementary Figure 25. XPS spectra of Pd/b2-TiO_2/b-Si photoelectrode after PEC water splitting for 100 h. **a** XPS survey spectrum for the surface of Pd/b2-TiO_2/b-Si. **b** Elemental depth profile of Pd/b2-TiO_2/b-Si. **c** Ti 2p spectrum for the sample surface. **d** Ti 2p spectrum for the inner of the sample after the etching depth of 20 nm.

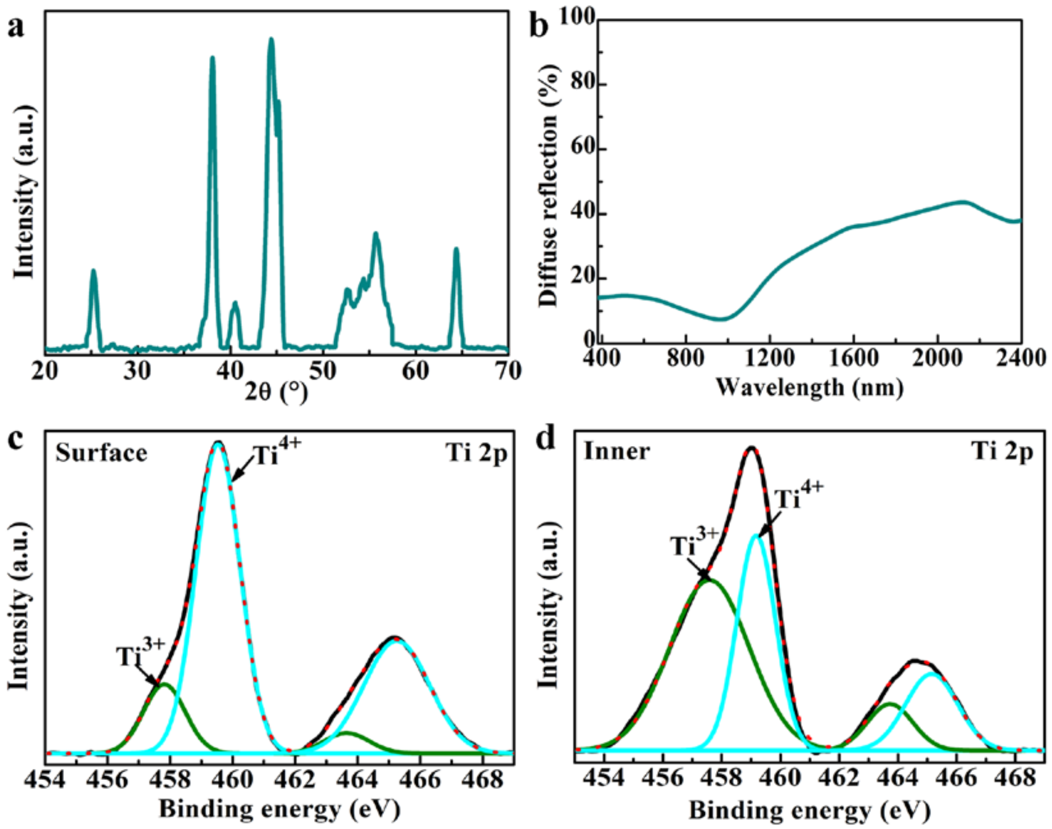

Supplementary Figure 26. The characterization of b2-TiO_2/planar Si. **a** XRD pattern of b2-TiO_2/planar Si. **b** The measured total hemispherical optical reflectance of b2-TiO_2/planar Si. **c** Ti 2p spectrum for the surface of Pd/b2-TiO_2/planar Si. **d** Ti 2p spectrum for the inner of Pd/b2-TiO_2/planar Si after the etching depth of 20 nm.

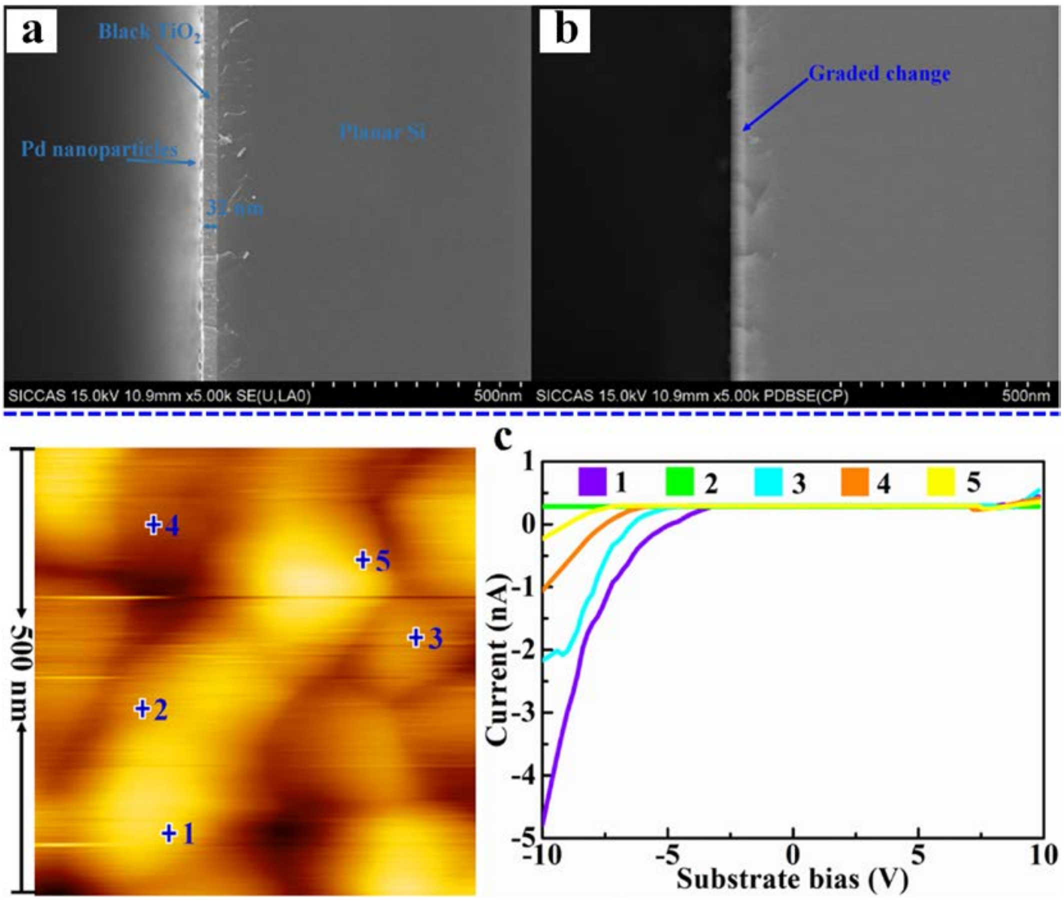

Supplementary Figure 27. The structure and conductive property of b2-TiO_2/planar Si. **a** Cross-sectional FESEM image of Pd/b2-TiO_2/planar Si. The scale bar labels are 500 nm. **b** The backscattered electron image corresponding to Supplementary Fig. 27a. **c** AFM topography image and typical *I*-*V* curves of marked position by the number for Pd/b2-TiO_2/planar Si. Position 1, 2, 3, 4 and 5 in AFM image corresponding to purple, green, Cambridge blue, orange and yellow *I*-*V* curve, respectively.

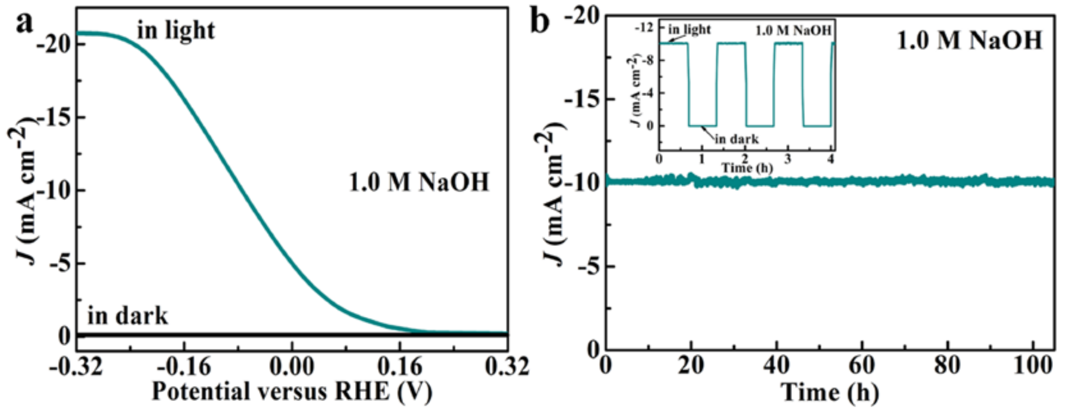

Supplementary Figure 28. PEC performance of Pd/b2-TiO_2/planar Si. **a** *J-V* curves of the sample (scan rate is 0.01 $V \cdot s^{-1}$) in 1.0 M NaOH under 1 sun illumination. The current density measured in dark is almost horizontal line, namely 0 $mA \cdot cm^{-2}$. **b** *J-t* plot of the sample held at -0.077 V vs RHE in 1.0 M NaOH under 1 sun illumination. The inset is the variations of photocurrent density at -0.077 V vs RHE by chopping light illumination.

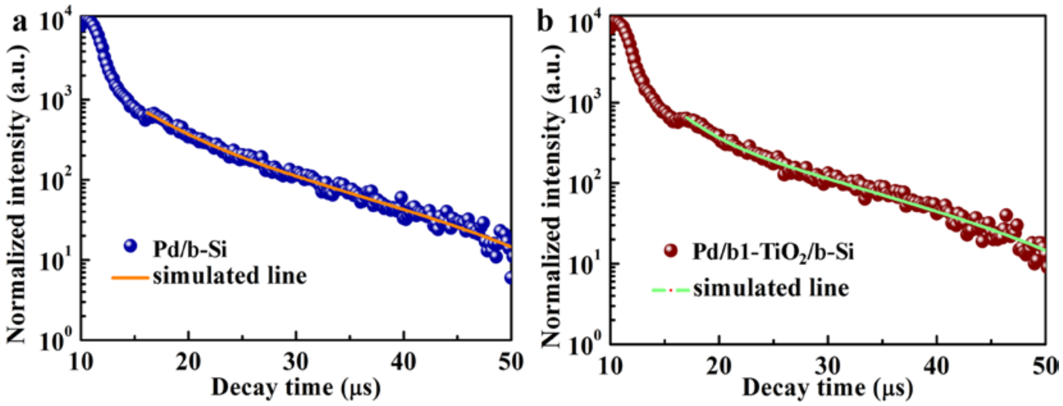

Supplementary Figure 29. Charge carrier dynamics of the samples. **a** Time-resolved PL decay curve of Pd/b-Si at room temperature. **b** Time-resolved PL decay curve of Pd/b1-TiO_2/b-Si at room temperature. Solid lines represent the kinetic fit using bi-exponential decay model.

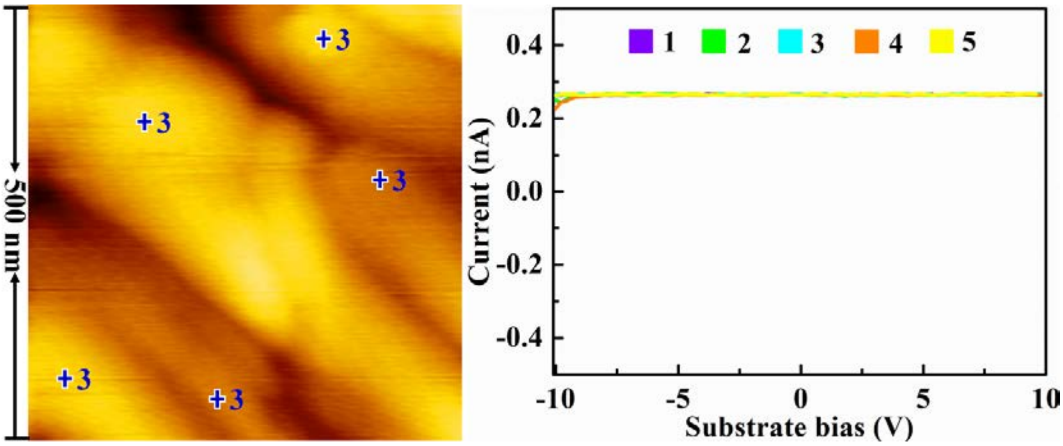

Supplementary Figure 30. AFM topography image and typical *I*-*V* curves of marked position by the number for Pd/c-TiO_2/b-Si. Position 1, 2, 3, 4 and 5 in AFM image corresponding to purple, green, Cambridge blue, orange and yellow *I*-*V* curve, respectively.

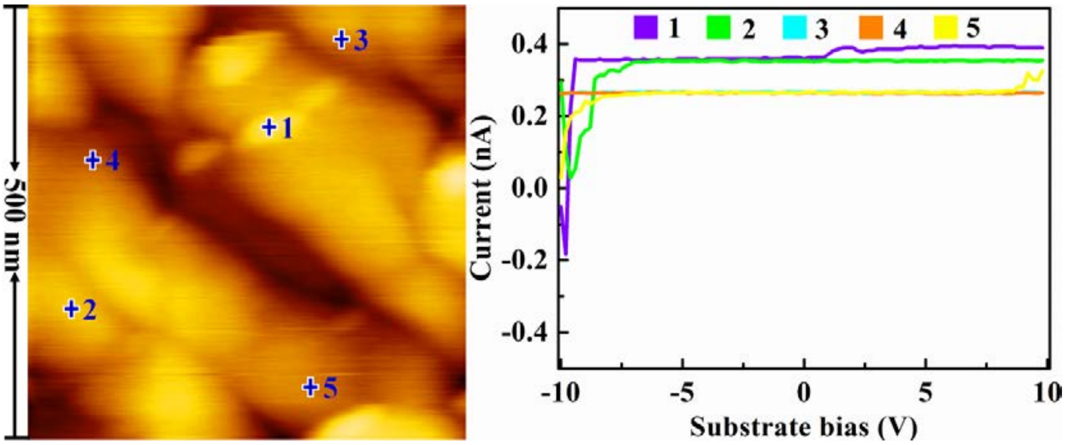

Supplementary Figure 31. AFM topography image and typical *I*-*V* curves of marked position by the number for Pd/b1-TiO_2/b-Si. Position 1, 2, 3, 4 and 5 in AFM image corresponding to purple, green, Cambridge blue, orange and yellow *I*-*V* curve, respectively.

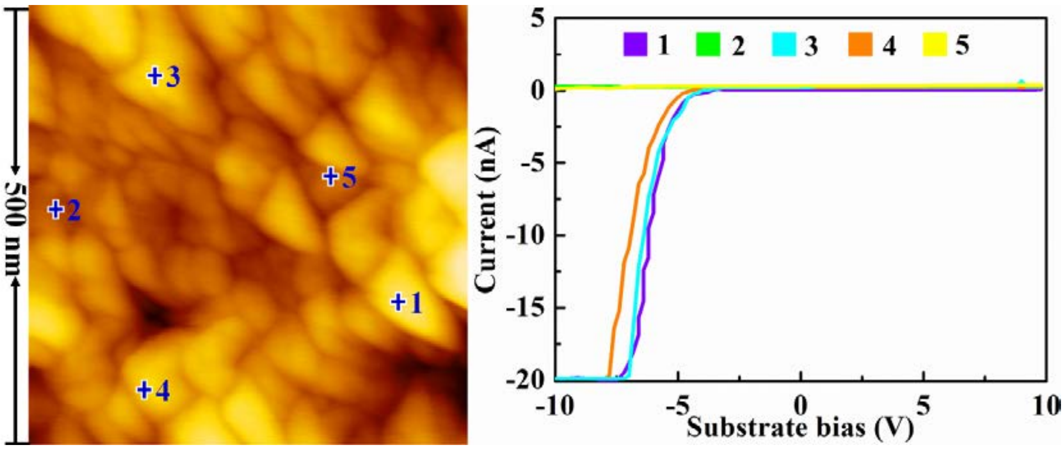

Supplementary Figure 32. AFM topography image and typical *I*-*V* curves of marked position by the number for Pd/b-Si. Position 1, 2, 3, 4 and 5 in AFM image corresponding to purple, green, Cambridge blue, orange and yellow *I*-*V* curve, respectively.

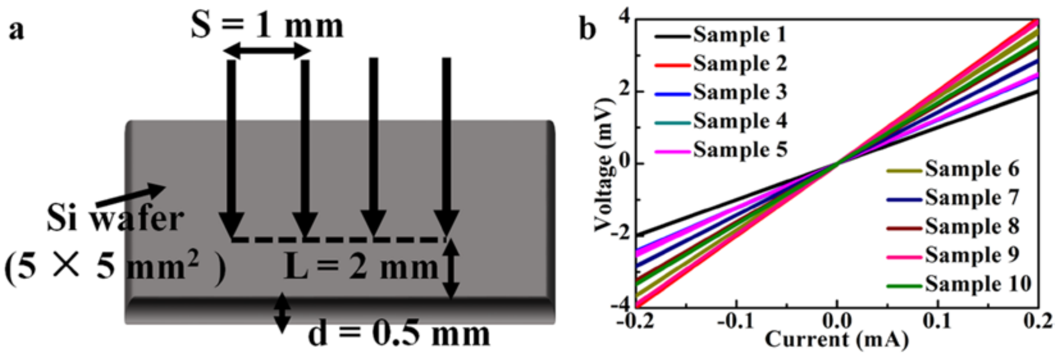

Supplementary Figure 33. The four probe method for measuring resistance. **a** Schematic of a four-point measurement with line-up tips forming the contacts on the surface of the planar Si. **b** Equidistant four-probe current-voltage curves of the planar Si.

As shown in Supplementary Fig. 33a, the thickness of Si wafer (d), the tip-tip distance (S) and the distance between tip and sample edge (L) are 0.5, 1 and 2 mm, respectively. The resistivity (ρ) of Si wafer is calculated as follows:

$$\rho = \frac{2\pi \mathrm{S}}{\mathrm{B}_0} \cdot \frac{V}{I} \tag{4}$$

where I is the applied current, V the corresponding voltage, S the tip-tip distance and B_0 the correction factor (3.104) determined by S/d and L/S. Based on the resistance (R) ranged from 10 to 20 Ω, the real resistivity of Si is around 2-4 Ω·cm.

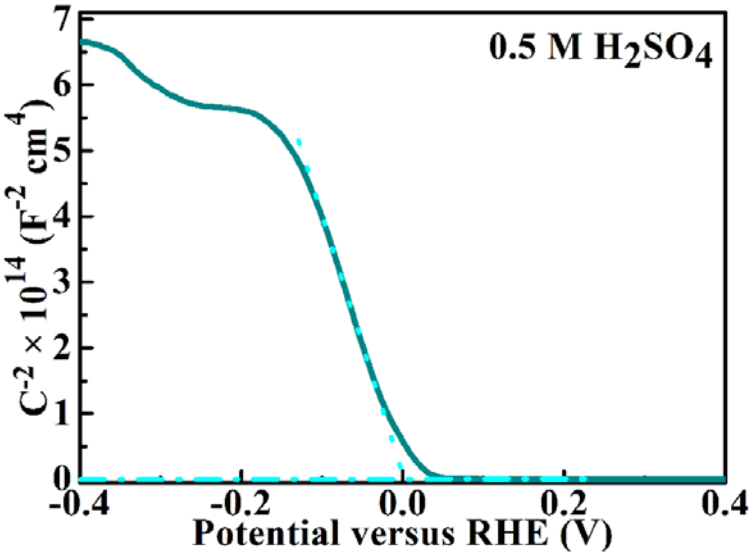

Supplementary Figure 34. Mott-Schottky plot of the planar Si from capacitance measurement as a function of potential vs RHE under dark conditions.

The Mott-Schottky plot were acquired at a frequency of 1 KHz in 0.5 M H_2SO_4 solution by a CHI 660 potentiostat. The Mott-Schottky equation is shown below:

$$\frac{1}{C^2} = \frac{2}{q\varepsilon_s \varepsilon_0 A^2 N_D}(V - V_{fb} - {kT}/{q}) \tag{5}$$

where C is capacitance, q the charge of an electron (1.60×10^{-19} C), ε_0 the vacuum permittivity (8.85×10^{-14} F·cm^{-1}), ε_s the permittivity of silicon (1.05×10^{-14} F·cm^{-1}), A the area of the sample, N_D the donor density, V the appied bias, V_{fb} the flat band voltage, k Boltzmann's constant (1.38×10^{-23} J·K^{-1}), and T the temperature (25 °C). The x-intercept of the Mott-Schottky plot was reached at the bias that needs to be applied to cause the bands to become flat. Also, the slope of the plot can be used to calculate the donor density of the electrode. The x-intercept plus kT/q (~0.025 V) equals the flat band voltage. The N_D can be calculated using the equation:

$$N_D = \frac{2}{e\varepsilon\varepsilon_0}\left[\frac{\mathrm{d}({1}/{C^2})}{\mathrm{d}V}\right]^{-1} \tag{6}$$

where ε and $\left[\frac{\mathrm{d}({1}/{C^2})}{\mathrm{d}V}\right]$ are the dielectric constant of silicon (11.68) and the slope of the sharp increase from 0-0.12 V region. Thus, N_D for the planar Si can be calculated to be 3.21×10^{15} cm^{-3}, corresponding to the resistivity of Si wafer (2-4 Ω·cm) basically.

Supplementary Table 1. Deposition of each layer to yield the cocatalyst/protective layer/semiconductor architecture.

Sample	Ti layer		TiO_2 layer		Pd layer	
	Power (W)	Time (min)	Power (W)	Time (min)	Power (W)	Time (min)
Pd/b-Si	−	−	−	−	20	2/3
Pd/c-TiO_2/b-Si	50	2/3	150	40	20	2/3
Pd/b1-TiO_2/b-Si	50	2/3	150	40	20	2/3
Pd/b2-TiO_2/b-Si	50	4/3	150	40	20	2/3

Supplementary Table 2. The kinetics parameters of Pd/b-Si, Pd/c-TiO_2/b-Si, Pd/b1-TiO_2/b-Si and Pd/b2-TiO_2/b-Si derived from time-resolved PL decay curves at room temperature.

Sample	t1	t2
Pd/b-Si	3.92 μs	13.34 μs
Pd/c-TiO_2/b-Si	0.16 ns	8.82 ns
Pd/b1-TiO_2/b-Si	2.72 μs	12.54 μs
Pd/b2-TiO_2/b-Si	4.07 μs	16.32 μs
